# Prunus mume extract and choline treatment in patients with non-alcoholic fatty liver disease estimated by b-mode ultrasonography and hepatorenal index

**DOI:** 10.22088/cjim.15.1.19

**Published:** 2024

**Authors:** Petar Avramovski, Miroslav Lazarevski, Maja Avramovska, Stefan Talev, Julijana Petrovska, Vesna Siklovska, Kosta Sotiroski

**Affiliations:** 1 Clinical hospital, Bitola, North Macedonia; 2 City General Hospital 8th September, Department of internal medicine, Skopje, North Macedonia; 3 Department of Obstetrics and Gynecology, Clinical Hospital Bitola, Bitola, North Macedonia; 4 Department of chirurgie, Clinical Hospital Bitola, Bitola, North Macedonia; 5 Department of clinical and interventional gastroenterology, Bitola, North Macedonia; 6 Department of radiology, Bitola, North Macedonia; 7 Department of Statistics, Ohrid University, Bitola, North Macedonia

**Keywords:** Prunus mume, Choline, Non-alcoholic fatty liver disease, Hepatorenal index, Functional liver tests, Ultrasonography

## Abstract

**Background:**

The aim of this study was to find the difference between the liver function test (LFT) and hepatorenal index (HRI), before and after the administration of *Prunus mume* (PM) and choline i.e., to find the predictors of the non-alcoholic fatty liver disease (NAFLD) severity according its HRI, during the three-month follow-up period.

**Methods:**

LFT, glucose, and lipid tests were determined in 168 NAFLD patients, at baseline and after three-month drug treatment. HRI was calculated by Image J software analyzing the ultrasound images, and according its value, 3 groups of NAFLD were formed.

**Results:**

The HRI at baseline (1.3598±0.1744) and after 3 months therapy (1.3061±0.1923) differs significantly (p<0.0001). Plasma glucose (FPG) (p<0.0001), glycated hemoglobin (HbA1c) (P=0.002), alanine aminotransferase (ALT) (p<0.0001), aspartate aminotransferase (AST) (P=0.0006), gamma-glutamil transferase (γ-GT) (P=0.0053), high density lipoprotein cholesterol (HDL-Ch) (p<0.0001) and triglycerides (P=0.041) differ significantly, too. HRI is positively correlated with: HbA1c (P=0.035), ALT (P=0.002), AST (P=0.003), γ-GT (P=0.043), and triglycerides (P=0.002) and inversely correlated with HDL-Ch (P=0.011). In multiple regression results (standard coefficient and p-value), the independent predictors for HRI in NAFLD patients were: HbA1c (0.1443, 0.0004), ALT (0.001142, 0.0081), triglycerides (0.0431, 0.0235) and γ-GT (0.001376, 0.0329).

**Conclusion:**

Three-month administration of PM and choline have beneficial effects on the regulation of glucose and lipid metabolism (HDL-Ch), and on LFT. This plant extract significantly reduces the levels of FPG, HbA1c, ALT, AST, γ-GT, triglycerides and increases HDL-Ch. The triglycerides, ALT, γ-GT and HbA1c are positive independent predictors for the severity of NAFLD.

Non-alcoholic fatty liver disease (NAFLD) defines a wide spectrum of histological abnormalities from fatty liver to nonalcoholic steatohepatitis (1) characterized by deposition of adipose tissue in the liver (2). NAFLD patients are at increased risk of liver-related diseases such as fibrosis, cirrhosis and hepatocellular carcinoma (2, 3). There is strong association between NAFLD and type 2 diabetes mellitus (T2DM) and endothelial dysfunction with increased risk of developing atherosclerosis which leads to coronary heart disease (3, 4). Many herbal extracts have antioxidant and anti-inflammatory activity and hepatoprotective effects [Silybum marianum, Curcuma longa, Taraxacum officinale, Prunus mume (PM), Ginkgo biloba, etc.] which have been previously demonstrated (5, 6). 

Aslam et al. reported a significant improvement of liver function tests (LFT) in 247 patients with abnormal bilirubin, alanine transaminase (ALT) and aspartate aminotransferase (AST) after treatment with PM and choline (7). PM, also known as Japanese apricot, is a herbal originating from south-eastern China with known hepato-protective, anti-inflammatory and antioxidant effect and potentially therapeutic effects for T2DM, vascular dementia, and hepato-cellular carcinoma (8).

The validity and accuracy of B-mode ultrasound in the detection of steatosis, based on characteristic liver sonographic findings (bright hepatic echoes, diffuse liver hyperechogenicity and enlarged liver with vascular blurring of the portal and hepatic vein) followed by a liver biopsy, was approved by Dasarathy et al.’s prospective study (9). An advanced B-mode ultrasound imaging method for the detection of NAFLD is based on the ratio of the average liver and kidney parenchyma echogenicity, which produce hepatorenal index (HRI) (10). Marshall et al. based on the results of strong correlation between HRI and histologic analysis of their study, concluded that HRI is an effective method for screening of patients with steatosis or abnormal LFT, especially for the patients who do not accept an invasive liver biopsy (10). The aim of this study was to find the difference and correlation between the HRI and LFT, before and after the administration of a fixed dose combination of PM extract with choline i.e., to find the independent predictors of the NAFLDs changes (by the HRI) during the three-month follow-up period.

## Methods

**Patients:** In this prospective longitudinal study, we enrolled 168 patients who came on abdominal B-mode ultrasound diagnostics during one year period and were preliminary selected from 450 consecutive patients, but only 168 were eligible for entry because they never consumed alcohol and had thicker subcutaneous tissue with a mean measurement greater than d = 25 mm (11).

 Patients under 18 years of age, the patients which were diagnosed before or medically treated for liver disease, severe heart disease and T2DM, the patients with obstructive jaundice, pregnant and lactating mothers, apart from a medical history, were excluded from the study. The patients with renal disease, with the presence of right kidney cortical scarring, hydronephrosis, or renal cysts, were also excluded. The exclusion criteria in our study were the similar as those of Marshall et al.’s study (10). The main selection criterion for entry was B-mode ultrasonographically confirmed diagnosis of steatosis, in accordance with ultrasound's criteria for it (12). All participants signed an informed consent and the study was approved by the Ethics Committee of our institution (code 28.08.2021/3).

Demographic data about the health condition and history of the patients' past disease were taken from the medical records and clinical register data to establish the criteria for possible exclusion of some of the patients. Patients were given one tablet LEVIKER^®^, *PM (standardized extract á 150 mg) and choline (á 82.5 mg*) preferably early in the morning with breakfast, continuously daily for three months. The patients agreed to take only tablets without other additional activities such as exercise or change in the hygienic-dietary regime. In this way, we perceive only the effects of LEVIKER^® ^with exclusion of the effects of changed lifestyle, which is more objective to show only the effects of its medicinal properties. Appropriate laboratory analyses as venous blood samples and HRI were measured before (baseline) and after 3 months drug treatment.

**Assessment:**

**B-mode ultrasonography:** We used B-mode ultrasonography to provide images of the liver and adjacent kidney. The sonographic examinations were performed by Logiq pro 5 ultrasound scanner (GE Medical Systems-USA: 4855WElectric Avenue, Milwaukee) with abdominal convex multi-frequency (1.8-5 MHz) ultrasound probe GE 4-SC. To get the best quality images with good deep focus, with proper brightness, contrast and resolution, manual adjustment mode of the machine was used (13). The distance "d" was ultrasonographically measured by placing of the caliper between the skin and the liver surface (11). Three ultrasonographers (radiologist, gastroenterologist and internist with over than 35 years’ experience) performed independent ultrasound examinations and "ImageJ" analysis. Interobserver reliability was determined using Cohen's kappa coefficient (κ) for estimation of the HRI (κ = 0.913).

**Figure 1:**
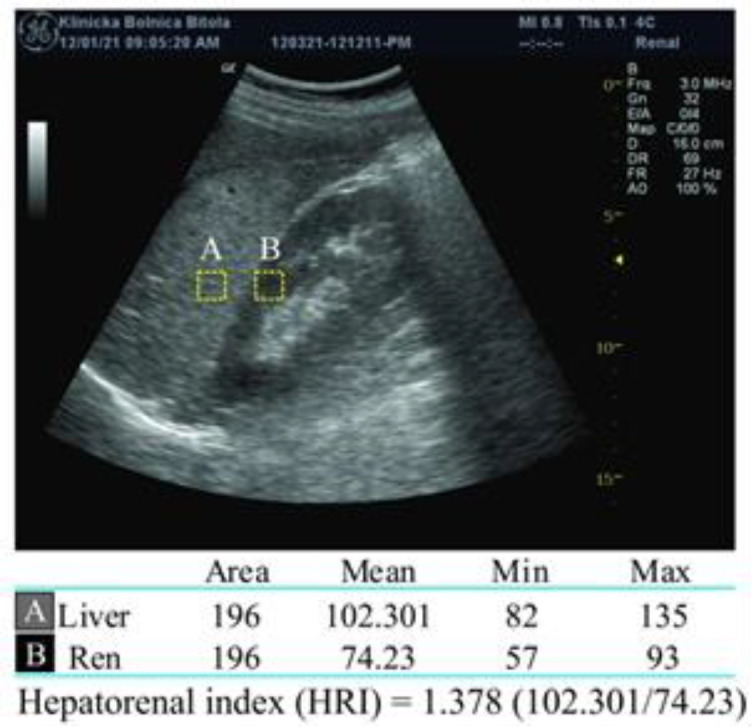
Analysis of an ultrasound image using ImageJ software and calculation of the hepatorenal index

**Image analysis obtained by ultrasonography:** We used "ImageJ", free software to analyze the images obtained ultrasonographically, (https://imagej.nih.gov/ij/download.html) which has been used and confirmed in several studies, previously (3, 12, 14, 15). This software compares the echogenicity of large number of points in the liver parenchyma with approximately the same number in the right kidney parenchyma. Each of the analyzed small dots from both parenchymas receives its own echogenicity gradation from 0 to 255 according to the gray scale. We analyzed 196 pixels with a measured mean value of 102.301 for the liver tissue (A) and mean value of 74.23 for the kidney tissue (B). After that, “A” was divided by “B” to calculate the HRI (102.301/74.23 = 1.378). The principle of analysis is shown in figure 1.

**Clinical and biochemical parameters:** After an overnight fast of at least ten hours, venous blood samples were collected for evaluation of biochemical parameters. Fasting plasma glucose (FPG), glycated hemoglobin (HbA1c), ALT, AST, gamma-glutamil transferase (γ-GT), cholesterol, high density lipoprotein cholesterol (HDL-Ch), low density lipoprotein cholesterol (LDL-Ch), triglycerides, total bilirubin and direct bilirubin were determined twice (at baseline and after 3 months) in all 168 participants using standard laboratory procedures performed on a Cobas Mira S Analyzer (Roche Diagnostics, Holliston, Massachusetts, USA). 

**Statistical analysis:** MedCalc Statistical Software Version 20.006 (MedCalc Software Ltd, Ostend, Belgium) was used for data analysis. The results were presented as median and 25^th^ to 75^th^ percentiles, mean and standard deviation (SD), number (N) and percentage (%). 

Student paired t-test, Wilcoxon signed-rank test or chi-square test were used appropriately to find the differences between baseline and after a 3-month data within a group. Partial correlation was used to find the strength and direction of the relationship between HRI and demographic and laboratory variables. Linear regression analysis between dependent and independent variables were performed to find their relationships. Multiple backward regression analysis was used to find the determinants of the dependent variable HRI. Receiver operating characteristics (ROC) curves and area under curves (AUC) was used to calculate the sensitivity and specificity of HRI in predicting NAFLD. A p-value less than 0.05 (two-tailed) considered a statistically significant difference.

## Results

**Demographic characteristics:** B-mode ultrasonography and appropriate laboratory LFT, glucose and lipid tests were successfully conducted on 168 patients from general populations aged 51.27±10.04 years (range 26-70), with their body mass index (BMI) of 26.45 kg/m^2^, 52 patients were smokers and 28 were hypertensive, estimated at baseline and after 3 months. The demographic and clinical characteristics of the patients are presented in table 1.

The statistical significance of the differences between pre-intake baseline results and results obtained after 3 months is represented by the p-value. We detected in 50 (29.8%) patients mild NAFLD (HRI = 1.1766±0.1744); 103 (61.3%) patients had moderate NAFLD (HRI = 1.3971±0.1111) and 15 (8.9%) patients had severe NAFLD (HRI = 1.7147±0.0511). There was a statistically significant difference (p < 0.05) between weight and the majority of laboratory test results obtained before and after drug treatment (FPG, HbA1c, ALT, AST, γ-GT, cholesterol, HDL-Ch, triglycerides and total bilirubin), except in LDL-Ch (P = 0.269) and direct bilirubin (P = 0.458). There was a statistically significant difference (p < 0.0001) between the HRI in total NAFLD patients, in mild and in moderate NAFLD group, but not between the HRI in severe NAFLD patients group (P = 0.361). 

The last column “Δ (%)” in the table 1 shows percentage decrease or increase of the examined variables during the 3-month follow-up period in the variables which showed a statistically significant difference before and after the applied drug therapy, only. HRI decreased significantly from 1.3598 to 1.3061 by 3.95%, p < 0.0001. Treatment response of PM on 168 patients show that only 6/168 (3.57%) patients do not respond in changing of LFT and hepatorenal index, especially the patients which belong in severe NAFL.

**Figure 2:**
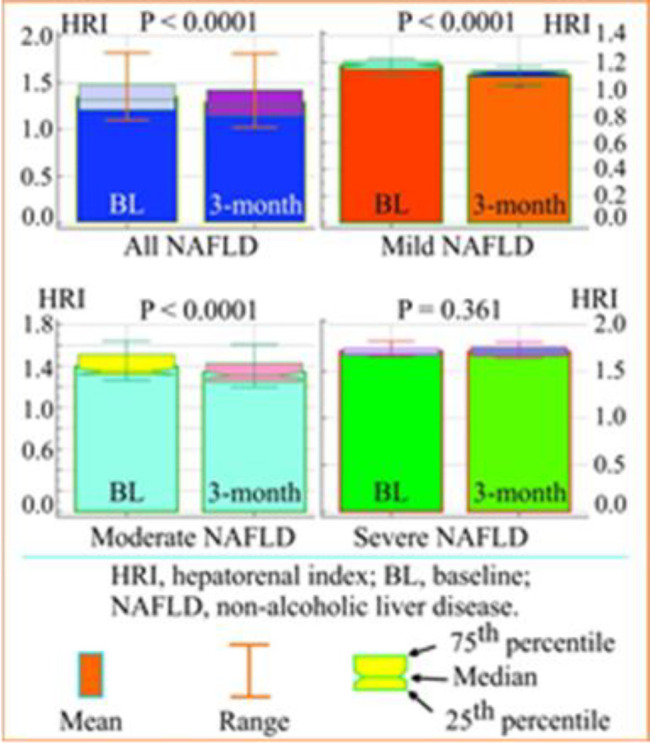
Paired samples t-test, box and whisker plot of the mean, range, median, and 25th and 75th percentiles of hepatorenal index at baseline and after 3-month period in non-alcoholic fatty liver disease patients (all patients and three subgroups)

**Comparison of the HRI at baseline and after three months:** The results of the HRI (mean, range, median and 25th and 75th percentiles) for all unclassified NAFLD patients, for mild, moderate and severe NAFLD are shown as comparative Box-and-Whisker plots in figure 2. The results of the p-value for the significance of the difference of the HRI between two estimated period were: for unclassified, mild and moderate NAFLD (p < 0.0001) and for the severe NAFLD patients (P = 0.361). 

**Partial correlation:** In partial correlation, at baseline, we found a statistically significant correlation between HRI and the following variables: HbA1c (P = 0.032), ALT, AST and γ-GT (P = 0.002, P = 0.003, P = 0.043, respectively), HDL-Ch (P = 0.011) and triglycerides (P = 0.002). There was no statistically significant correlation between HRI and other variables at baseline. The results are presented in table 2.

**Figure 3:**
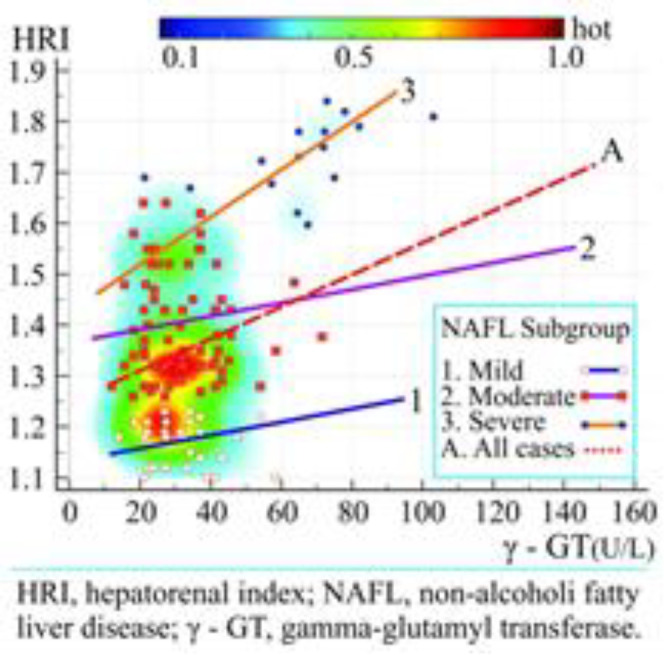
Heat scatter plot and linear regression lines represent the relationship between the hepatorenal index and Gamma-glutamyl transferase in different non-alcoholic fatty liver disease groups

**Linear regression and heat scatter plot:** The heat scatter plot of the linear regression analysis between HRI and γ-GT in three different NAFLD groups [mild (blue line 1), moderate (purple line 2) and severe (orange line 3) and all NAFLD patients group (red dashed line A) is shown on figure 3.

**Table 1:** Demographic and clinical characteristics of the patients at baseline and after 3-month follow-up period

**Characteristics (N = 168)**	**Baseline**	**After 3-months**	**Test**	**P-value**	**Δ (%)**
**Age (years)**	51.27 ± 10.04	51.77 ± 10.04	0.456	0.648	/
**Height (cm)**	172.0 (165.0 - 183.75)	172.0 (165.0 - 183.75)	/	/	/
**Weight (kg)**	78 (72.0 - 86.75)	77 (71 - 85.7)	-2.1366	0.033	-1.28
**Body mass index (kg/m** ^2^ **)**	26.45 (23.31 - 28.08)	26.30 (23.2 - 27.89	-1.9167	0.055	/
**Hypertension, N (%)**	28 (16.6)	31 (18.4)	0.188	0.665	/
**Smokers, N (%)**	52 (30.9)	49 (29.2)	0.115	0.734	/
**Fasting plasma glucose (mmol/L)**	5.69 ± 0.56	5.11 ± 0.71	8.3135	<0.0001	-10.19
**Glycated hemoglobin (HbA1c) (%)**	5.81 ± 0.53	5.65 ± 0.42	3.0667	0.002	-2.75
**ALT (U/L)**	72.3 ± 20.2	47.6 ± 17.6	11.9495	<0.0001	-34.16
**AST (U/L)**	54.5 ± 17.9	48.3 ± 14.7	3.4695	0.0006	-11.38
**γ-GT (U/L)**	36.17 ± 12.8	32.5 ± 11.1	2.8076	0.0053	-10.14
**Cholesterol (mmol/L)**	5.61 ± 1.05	5.32 ± 1.04	2.5434	0.011	-5.17
**HDL-Ch (mmol/L)**	1.39 ± 0.19	1.56 ± 0.21	7.7807	<0.0001	+12.23
**LDL-Ch (mmol/L)**	3.38 ± 0.95	3.27 ± 0.87	1.1068	0.269	/
**Triglycerides (mmol/L)**	2.54 ± 1.12	2.34 ± 0.93	2.0478	0.041	-7.87
**Total bilirubin (mmol/L)**	14 (8 - 23)	13 (7 - 21)	3.3144	0.00046	-7.14
**Direct bilirubin (mmol/L)**	5 (3 - 7)	5 (3 - 7)	-0.10532	0.458	/
**Hepatorenal index (HRI)**	1.3598 ± 0.1744	1.3061 ± 0.1923	-29.292	<0.0001	-3.95
**HRI - mild; 50 (29.8)**	1.1766 ± 0.0381	1.1094 ± 0.0405	-36.965	<0.0001	-5.71
**HRI - moderate; 103 (61.3)**	1.3971 ± 0.1111	1.3427 ± 0.1223	-29.997	<0.0001	-3.89
**HRI - severe; 15 (8.9)**	1.7147 ± 0.0511	1.7107 ± 0.05337	-0.945	0.361	/

ALT, Alanine Aminotransferase; AST, Aspartate Aminotranserase; γ-GT, Gamma-glutamyl transferase; HDL-Ch,

high density lipoprotein cholesterol; LDL-Ch, low density lipoprotein cholesterol; NAFLD, non-alcoholic fatty liver

disease; HRI, hepatorenal index; Δ (%), delta percentage changes from the baseline to the third month.

Criteria for NAFLD: mild (HRI = 1.05 - 1.24), moderate (HRI = 1.25 - 1.64) and severe (HRI) ≥ 1,65. The results are expressed

as: mean ± SD (standard deviation), or median and (25th to 75th percentiles) and N (%), number and percent.

**Table 2:** Partial (r_p_) correlation between hepatorenal index and estimated parameters at baseline

**Variables**	**Hepatorenal index**	
**Demographic and laboratory**	**r**_p_	**P**
**Age (years)**	0.072	0.354
**Body mass index (kg/m** ^2^ **)**	0.029	0.709
**Hypertension, N (%)**	0.119	0.124
**Smokers, N (%)**	0.086	0.267
**Fasting plasma glucose (mmol/L)**	0.146	0.068
**Glycated hemoglobin (HbA1c) (%)**	0.165	0.032
**ALT (U/L)**	0.241	0.002
**AST (U/L)**	0.229	0.003
**γ-GT (U/L)**	0.156	0.043
**Cholesterol (mmol/L)**	0.054	0.486
**HDL-Ch (mmol/L)**	-0.195	0.011
**LDL-Ch (mmol/L)**	0.141	0.068
**Triglycerides (mmol/L)**	0.241	0.002
**Total bilirubin (mmol/L)**	0.069	0.374
**Direct bilirubin (mmol/L)**	0.084	0.279

rp, Partial correlation coefficient;

ALT, Alanine Aminotransferase; AST, Aspartate Aminotransferase;

γ-GT, Gamma-glutamyl transferase; HDL-Ch, high density lipoprotein cholesterol;

LDL-Ch, low density lipoprotein cholesterol.

**Figure 4:**
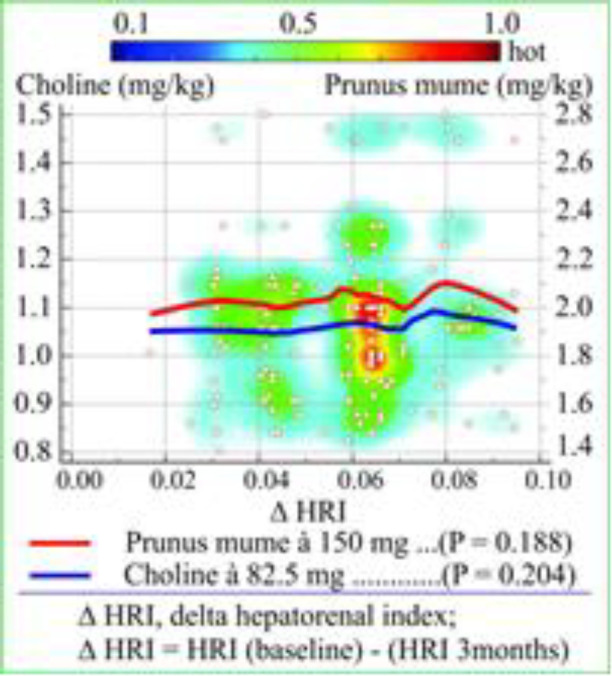
Heat scatter plot and linear regression lines represent the correlation between the Δ hepatorenal index and the dose of Choline and Prunus mume in non-alcoholic fatty liver disease patients

The equation of linear regression analysis (y = 1.2758 + 0.001616·x) represents the relationship between a scalar dependent variable HRI and an explanatory variable γ-GT. The regression parameter b_1_ (0.001616) signified that with each increase of unit in γ-GT (U/L), the HRI score increased by 0.001616. The results of analysis of variance (ANOVA) showed a statistically significant level for predicting dependent variable HRI by independent variable γ-GT (P = 0.0211, R^2^ = 0.03474). The average administered doses (mg/kg) of the prescribed drugs PM á 150 mg and Choline á 82.5 mg were obtained by dividing the dose (mg) with body mass (kg). Delta HRI (ΔHRI) is decrease of HRI after the 3-month drug treatment [HRI (baseline) - HRI (3-months)]. The median values of the administered doses of PM and Choline and Δ HRI were as follows: 1.973 mg/kg and 1.058 mg/kg, and 0.0595, respectively. The heat scatters plot and correlation regression lines of the administered dose with ΔHRI were shown in figure 4. There is no statistically significant correlation between the drug administered dose and Δ HRI (P = 0.188, P = 0.204). 

**Figure 5:**
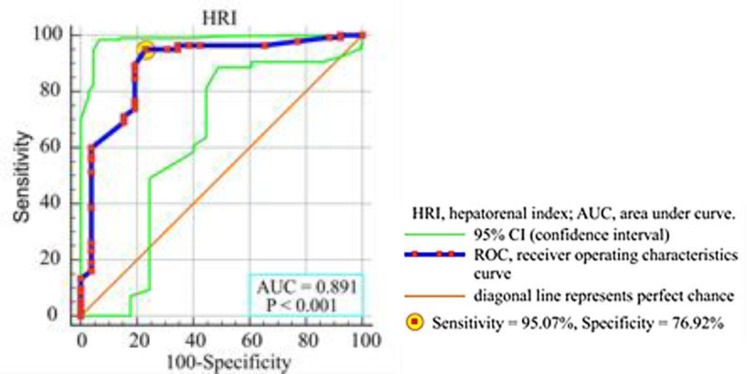
Receiver operating characteristics of HRI in predicting non-alcoholic liver disease with different percent of fatty mass accumulation

**Receiver operating characteristics (ROC) curve:** We calculated the sensitivity (95.07%) and specificity (76.92%) of the HRI in predicting the NAFLD, and we calculated the optimal cutoff point of HRI (> 1.31) to predict the NAFLD. Selecting our patients according to grayscale, we divided them in two groups, steatosis identified as fat less than 5% (category 0) and fat greater than 5% (category 1), and we calculated area under curve (AUD) = 0.891 (p < 0.001), Youden index J = 0.7199. Figure 5 shows ROC curve and AUC in predicting NAFLD by the HRI. The accuracy of the test depends on how well the test (HRI) separates the group into those without or with the disease (fatty greater than 5%). 

**Multiple regression analysis:** The results of a multiple backward regression analysis which show the predictable values of independent variables, HbA1c, ALT, triglycerides, γ-GT (as well as: cholesterol, FPG, AST, HDL-Ch, LDL-Ch, total bilirubin and direct bilirubin - rejected according to the model's criteria) on the dependent variable Y (HRI) are shown in table 3. Based on coefficient of elasticity η value (0.03660) we predict that HRI will increase by 3.66% for every 1% increase of γ-GT. There was a positive correlation between HRI and HbA1c, HRI and ALT, HRI and triglycerides and HRI and γ-GT. The coefficient of determination R^2^ (0.2189) showed that 21.89% from HRI in predicting of the NAFLD was dependent on predictive variables (input variables) HbA1c, ALT, triglycerides and γ-GT. The p-value followed the order of statistical significance: HbA1c (0.0004), ALT (0.0081), triglycerides (0.0235) and γ-GT (0.0329) 

**Table 3:** Multiple backward regression analysis of determinants of hepatorenal index

**Multiple regression**							
Dependent Y		**Hepatorenal index**					
**Least squares multiple regression**							
Method: Backward		Enter variable if P < 0.05; Remove variable if P > 0.5					
Sample size		168					
Coefficient of determination R^2^		0.2189					
Multiple correlation coefficient		0.4679					
**Regression Equation**							
**Independent variables**	**Coefficient**	**Std.** **Error**	**t**	**r **_partial_	**η**	**VIF**	**P**		
(Constant)	0.3825								
**Glycated hemoglobin** **(HbA1c) (%)**	0.1443	0.0395	3.653	0.286	0.6165	1.133	0.0004	
**ALT (U/L)**	0.001142	0.0004	2.686	0.214	0.0607	1.084	0.0081	
**Triglycerides (mmol/L)**	0.0431	0.0188	2.288	0.184	0.0805	1.186	0.0235	
**γ-GT (U/L)**	0.001376	0.0006	2.153	0.173	0.0366	1.039	0.0329	
**Cholesterol (mmol/L)**	-0.008404	0.0100	-0.836	-0.068	-0.0346	1.109	0.4047	

Variables not included in the model: Fasting plasma glucose, Aspartat Aminotransferase, High Density

Lipoprotein Cholesterol, Low Density Lipoprotein Cholesterol, Total bilirubin and Direct bilirubin.

Std. Error, standard error; VIF, Variance Inflation Factor; ALT, Alanine Aminotransferase;

γ-GT, Gamma-glutamyl transferase; η, (eta) coefficient of elasticity.

## Discussion

To the best of our knowledge, this is the first prospective study that investigates the therapeutic effect of PM and Choline in NAFLD patients using HRI obtained by B-mode ultrasonography and LFT assessment over a three-month follow-up period. The study of Aslam et al. is closest to the objectives of our study, although our study assessed the efficacy of three-month therapy with PM and Choline, not only through the established difference in the results of FLT, but also through the measured HRI difference in patients with NAFLD, before and after the drug treatment (7).

At the end of this study, FPG, HbA1c, serum ALT, AST, γ-GT, total cholesterol, triglycerides and total bilirubin levels significantly decreased compared with pre-intake baseline levels, with the exception of HDL-Ch levels which significantly increased (table 1). The results of this study also reported similar findings as findings in Aslam et al.’s study where statistically significant decrease in the levels of ALT (p< 0.01), AST (p< 0.01) and total bilirubin (P = 0.04) were reported in 247 patients with abnormal levels of LFT (7). Similarly, Beretta et al. in their double-blind, placebo-controlled study in 45 healthy volunteers, reported significantly decreased levels in percentage of FPG (-11%), ALT (-47%), AST (-7%), γ-GT (-15%) and triglycerides (-8%) and an increase in HDL-Ch levels by 13% after 3 months PM treatment versus baseline, in accordance with our study (16). The significant percentage changes “Δ (%)” in LFT and lipid status before the mentioned studies are consistent with the percentage changes of biomarkers in our study. The studies are also consistent with the fact that the drug has no effect on changes in LDL-chin, the three-month period studied. However, the comparative results and the results of our study reliably confirm the effect of PM and Choline on the regulation of lipid and glucose metabolism and aminotransferases activity, as well as the effect on the decrease of γ-GT (17, 18).

Choline deficiency as a cause of alteration in choline and phospatidylcholine metabolism has an impact on pathways that predispose fatty liver. A secondary manifestation of its deficiency is an increase in triglyceride synthesis (18). Nakamura et al**.** discovered the association between accumulation of liver lipids and Choline deficiency 55 years ago (19). We found no statistically significant decrease of HRI during 3 months supplementation among the patients with severe NAFLD (P = 0.361). Possible explanation for this finding could be related to the advanced substitution of fat for fibrosis during NAFLD progression i.e. HRI is not reliable enough for grading severe steatosis with advanced liver fibrosis (20, 21). Besides, in our study, HRI was proven to be a very reliable marker in predicting the level of fatty accumulation with sensitivity of 95.07% and specificity of 76.92% which is according the study of Marshal et al. (10). They found similar results (sensitivity of 100% and specificity of 54%) in their study on 101 patients with HRI cutoff value of 1.28. 

A crucial effect of the administration of this fixed dose combination is an increase of HDL-Ch which has a significant cardioprotective effect and it is prerequisite in reducing the percentage of fatty infiltration in NAFLD, resulting in an echogenicity decrease of liver parenchyma and thus a consequent decrease of HRI (21, 22). We found a statistically significant partial correlation between the HRI (as measure of the severity of NAFLD) and various glucose (HbA1c) and lipid-related biomarkers (triglycerides and HDL-Ch), also and between the HRI and LFT (ALT, AST and γ-GT), only. In the overweight patients (BMI ≥ 25 kg/m^2^), the HRI showed a statistically significant correlation with the BMI. The overweight subjects had a 3.5-fold increased risk for liver steatosis and NAFLD (23). Tutunchi et al. found that severity of liver steatosis significantly correlated with serum TG (P = 0.026), ALT (p < 0.001), AST (p <0.001), TG (P = 0.026) and FPG (P = 0.041), but not correlated with total cholesterol (P = 0.271) and LDL-Ch (P = 0.341), of which the results were very close to our study results (22).

Kasapogly et al. found a strong positive correlation between γ-GT and NAFLD degree (23). Elevated γ-GT level has been reported to be of prognostic significance of the severity of NAFLD, but also in prognostic significance in coronary artery disease (24, 25). Reduction of γ-GT under hepatoprotective therapy would have a beneficial effect of reducing NAFLD degree and reducing of cardiovascular disease risk. Γ-GT has high sensitivity and specificity for the diagnosis and prognosis of liver disease with a value greater than its simple association with biliary disease and ALT, AST and alkaline phosphatase (25, 26).

The different color linear regression lines (figure 3) can be used to predict the severity of the NAFLD (estimated by HRI) in each of NAFLD subgroup by the known γ-GT. Beretta et al. examined the efficacy of standardized extract of PM in liver protection during a 3-month treatment period in two groups: the first treated was with a low dose of 150 mg, and the second with a double dose of 300 mg (16). The high dose group did not show any significant decreases in ALT, AST and γ-GT, which differed from the decreases in levels of these enzymes in low dose group. The results of our study are the same: we did not find a significant improvement of NAFLD severity (Δ HRI) which depends on the average value of administered dose of the drug (PM and Choline) because there is no statistically significant correlation between the administered dose and Δ HRI (figure 4).

 In backward multiple regression analysis, we found that HbA1c, triglycerides, ALT and γ-GT are independent predictors with positive correlation with HRI as marker for the severity of NAFLD, but HbA1c plays superior role as strongest independent predictor (P = 0.0004). Yu et al. in their cross-sectional study performed on 4826 healthy patients found that HbA1c was in strong and independent correlation with increased risk for advanced fibrosis in NAFLD patients without diabetes, which is consistent with the results of our study. HbA1c level and insulin resistance had a strong correlation with NAFLD, independently of other metabolic components and obesity (26, 27). 

A major underlying pathophysiological mechanism in the genesis of NAFLD is insulin resistance because of increased gluconeogenesis and glycogenolysis in the liver (27). PM reduces cellular oxidative stress through inhibition of mitogen-activated protein kinase’s (MAPK) activation and p-53 and p38 mediated apoptosis signalling pathway, thereby reducing liver-steatosis, inflammation and apoptosis (7). Its action includes hepato-protective activity, antioxidant/anti-inflammatory activity, boosting HDL cholesterol as well as lowering glucose levels, elevating liver function generally and avoiding metabolic and inflammatory-based illnesses (7, 26). That suppresses lipogenesis in liver tissue by inhibition of MAPK's activation. Suppression of de novo fatty acids production and enhanced non-esterified fatty acid release from adipose tissue (lipolysis) caused by PM result in a reduction of liver steatosis and in the reduction in its progression (27). Reduced muscle’s glucose disposal with consequent hyperinsulinemia, deregulated lipogenic factors and activated hepatic lipogenesis, additionally contribute of NAFLD development (27). 

Tomizawa et al.’s study indicates that NAFLD is more correlated with hypertriglyceridemia compared to hyper-LDL cholesterolemia and hypo-HDL cholesterolemia (28). They found by stepwise analysis in 168 patients, that triglyceride is predictor of NAFLD in comparison with FPG and HbA1c. In our study, triglyceride may slowly but surely take the leads as independent predictor of NAFLD, step by step with γ-GT and ALT, but certainly leaving the lead to HbA1c. However, it is still debatable whether triglyceride is a cause, consequence, or mere marker of hepatocyte injury. Banderas et al. studied 193 nondiabetic individuals with metabolic syndrome and NAFLD (29). They found that the main factors that predict the occurrence of the NAFLD as a common complication of metabolic syndrome are the levels of blood triglycerides and γ-GT, as well as obesity. In our study, the severity of NAFLD (HRI) will increase by 3.66% and 8.05% for every 1% increase of γ-GT or triglycerides, respectively.

The previously proven antioxidant, anti-inflammatory and hepatoprotective effect of PM and choline is manifested as improve of the LFT, glucose metabolism and lipid-related biomarkers leading to a decrease in the NAFLD severity measured by the HRI.

The first limitation is the relatively small number of patients, especially in the group of severe NAFLD. The second limitation is a relatively short follow-up period to detect significant reductive changes in the liver parenchyma in severe NAFLD patients, although this period is sufficient to highlight changes in biochemical functional tests.

PM and Choline (Leviker®) have beneficial effects on the regulation of the glucose and lipid metabolism, liver protecting property and in the increase of the HDL-CH. Quarterly treatment with this plant extracts significantly reduces FPG, HbA1c, ALT, AST, cholesterol, triglycerides, total bilirubin and γ-GT as surrogate marker for NAFLD, which consequently reduces the severity of NAFLD and HRI. 

The triglycerides, ALT and γ-GT are positive independent predictor and HbA1c is strong positive predictor for the severity of the NAFLD, and they can be used in the NAFLD diagnosis and prediction of its prognosis. There is no significant improvement in the severity of NAFLD which depends on the different average value of administered dose of PM and choline.
